# Using Co-design With Breast Cancer Patients and Radiographers to Develop “KEW” Communication Skills Training

**DOI:** 10.3389/fpsyg.2021.629122

**Published:** 2021-02-22

**Authors:** Mara van Beusekom, Josie Cameron, Carolyn Bedi, Elspeth Banks, Rachel Harris, Gerry Humphris

**Affiliations:** ^1^Population and Behavioural Sciences, School of Medicine, University of St Andrews, St Andrews, United Kingdom; ^2^Edinburgh Cancer Centre, Western General Hospital, Edinburgh, United Kingdom; ^3^National Cancer Research Institute, London, United Kingdom; ^4^Society and College of Radiographers, London, United Kingdom

**Keywords:** recurrence fear, psycho-oncology, radiotherapy, co-design, patient involvement, breast cancer, communication training

## Abstract

Previous work (FORECAST) has shown that concerns of breast cancer patients after finishing radiotherapy are responsive to conversations with radiographers during the treatment period. This study seeks to further understand radiographer and patient experiences, determine shared priorities for improvement in clinical interaction and develop communication guidelines and training to help radiographers support patients.

**Methods:** Using the principles of Experience-Based Co-Design, semi-structured interviews were held with *N* = 4 patients (videoed) and *N* = 4 radiographers, followed by feedback events (*N* = 7) to validate findings. Patients and radiographers exchanged experiences in a joint co-design session, agreed with shared priorities and generated ideas for further support. A survey was conducted for process evaluation. To scale up findings, UK-wide representatives from patient networks (*N* = 8) and radiographers and managerial staff (*N* = *16)* provided consultative input utilizing an iterative, adaptive procedure.

**Results:** Radiographers expressed a need for support with “difficult conversations,” especially those on Fear of Cancer Recurrence, and their appropriate management. Important pointers for reassuring communication were identified, including: being treated like a person, knowing what to expect, and space to ask questions. The co-design process was rated positively by both staff and patients. Thematic collation of findings and mapping these on literature evidence resulted in the “KEW” communication guidelines for radiographers: Know (Confidence; Expectations; Person), Encourage (Emotions; Space; Follow-up), Warmth (Start; Normalize; Ending). National stakeholder consultations validated and helped fine-tune the training model. The resulting training package, included: trigger videos (*n* = 6), a simulated patient scenario and interactive handouts on fears of cancer recurrence and the patient pathway.

**Conclusions:** The co-design process captured good practice to help standardize quality in empathic communication in the radiotherapy service. The resulting KEW: Know, Encourage, Warmth guidelines, and training package are user-centered as well as evidence-based. Supplementing single-site co-design with national consultative feedback allows for the development of interventions that are relevant to the clinical practice, even in detail, and helps to generate appropriate buy-in for roll out on a wider scale after evaluation.

**Trial Registration:** www.ClinicalTrials.gov ID: NCT03468881

## Introduction

While medical outcomes for people with a breast cancer diagnosis are steadily improving (Allemani et al., 2018), cancer patients' psychological needs often remain unmet (Sanson-Fisher et al., 2000). One of the most prevalent concerns relates to the possibility of the cancer coming back or progressing to other parts of the body (Fear of Cancer Recurrence), experienced by up to 86% of breast cancer survivors. At higher levels, such worries can lead to intense psychological emotional reactions, poor quality of life, and functional impairments (Simard et al., 2013).

The original FORECAST study, in which breast cancer patients' fears of cancer recurrence trajectories were mapped throughout and following their radiotherapy treatment, showed that patients are likely to experience lower levels of emotional distress after treatment if they get the space to express their concerns to their therapeutic radiographers while they are in receipt of the radiotherapy service (Barracliffe et al., 2018; Humphris et al., 2019). Although radiographers regularly encounter questions about emotional concerns both during these review sessions and at the treatment machine (Barracliffe et al., 2018), in the UK, there are no professional competencies associated with communication skills training (Azevedo et al., 2019).

A recent literature review showed that Communication Skills Training (CST) for the radiotherapy team has the potential to improve communication of staff members as well as patient outcomes such as anxiety and concerns (van Beusekom et al., 2019). There are also promising indications that training can help to improve the supportive skills of members of the radiotherapy team (Timmermans et al., 2006; Merckaert et al., 2015) and how often “emotional words” are used by patients when interacting with the trained radiotherapy team (Gibon et al., 2013; Merckaert et al., 2015).

A requirement for successful embedding of communication training in the context of radiotherapy is support from the organization and staff members who receive the training and being able to work around practical constraints within the service (Gibon et al., 2013; Liénard et al., 2016). A collaborative approach to developing such training together with relevant stakeholders can help to ensure a good fit with the day-to-day reality of the service and increase the likelihood of long-term engagement (Steen et al., 2011), a method described as co-design (Sanders and Stappers, 2008). Experience-Based Co-Design^1^ is an approach to co-design in the field of healthcare that encourages an exchange of experiences between patients and healthcare staff (Donetto et al., 2014, 2015; Rohde et al., 2016) to prioritize and work out improvements for a service.

This study describes the development of a model for empathic communication in the radiotherapy setting and corresponding communication skills training for radiographers. It builds upon the findings of the original FORECAST project, but uses a co-design approach to start with an in-depth qualitative understanding of radiographer and patient experiences and of shared priorities for quality in clinical interaction, to ensure the development of a user-centered, clinically relevant intervention to support the patient pathway.

## Materials and Methods

This paper covers phases 1 and 2 of the FORECAST2 study (van Beusekom M. M. et al., 2018): a co-design process with patients and staff and development process with national stakeholders input. The work has been approved by the London-Surrey Research Ethics Committee (18/LO/0669) and the University Teaching and Research Ethics Committee (MD13914). Informed written consent was obtained from all participants. All described sessions were facilitated by a health behavior expert (MB) and/or clinical psychologist with expertise in psycho-oncology (GH). Interactive and creative tools were used, designed to foster an open exchange between patients and staff. Materials included: portable white board and markers, “sticky note” pads, colored felt pens, video camera, handouts, index cards, and feedback grids.

### Experience-Based Co-design

**Figure 1:**
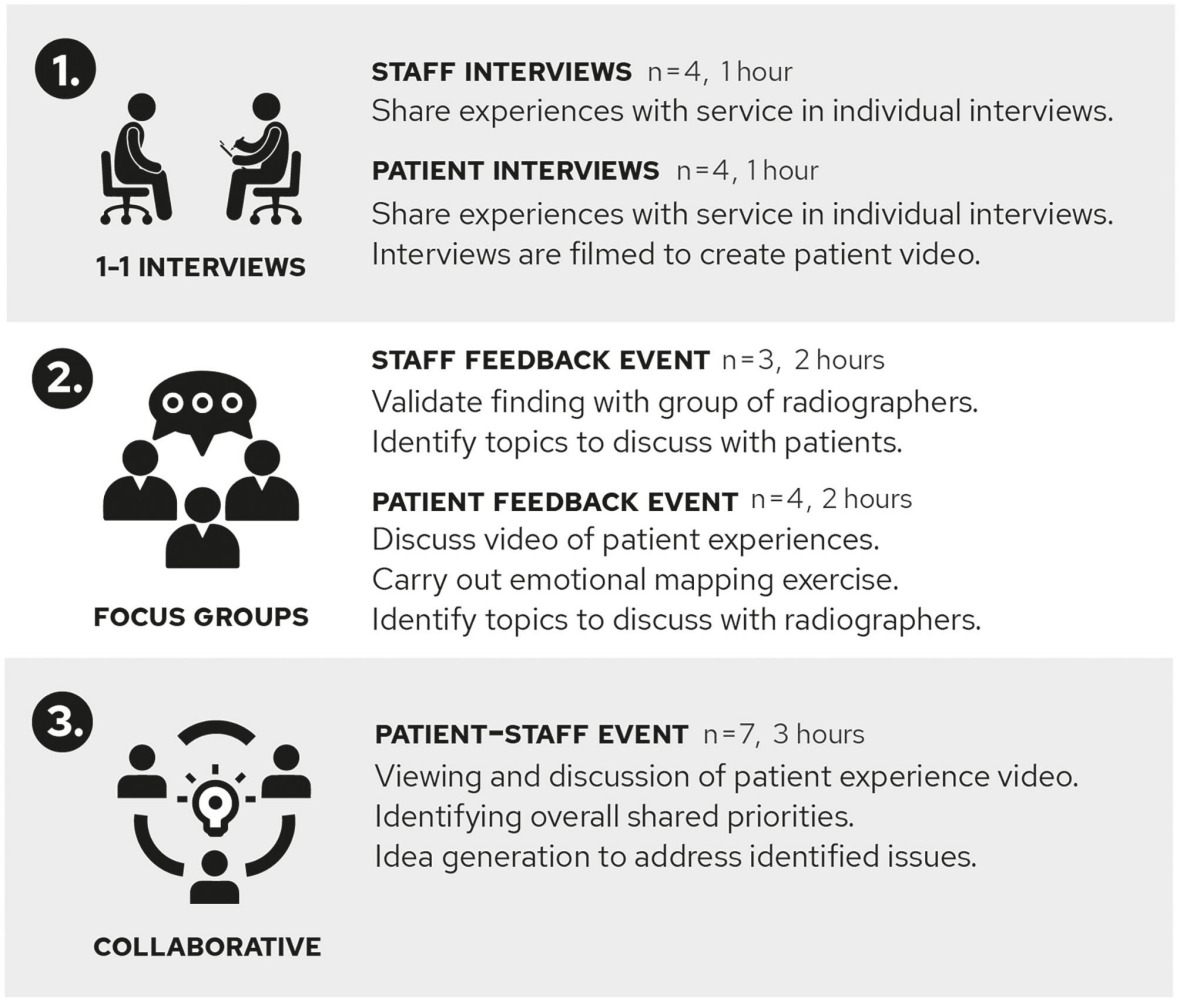
The three steps of the Experience-Based Co-Design Approach.

Experience-Based Co-Design (EBCD) principles and the online EBCD toolkit^2^, were used to capture how radiographers and patients with breast cancer experience the radiotherapy service, understand what helps to make a good experience and what could be further improved to support the patient experience (Figure 1).

#### Individual Interviews

First, semi-structured interviews were held (MB) with *N* = 4 radiographers (female; aged 32–48; 5–10 years or >10 years experience as therapeutic radiographer) and *N* = 4 women (aged 51–70; education from secondary education to University degree; no chemotherapy; no trastuzumab), who had recently finished radiotherapy for breast cancer at a major NHS Lothian Cancer Center. Patients were recruited and consented by the Clinical Radiographer Specialist on the project team and endorsed by responsible consultant. The 30- to 60-min-long interviews were filmed and used the topic guide shown in Table 1. Throughout the interviews, the researcher (MB) summarized the conversations to help validate findings.

**Table 1:** Topic guide for radiographer and patient individual interviews as part of the Experience-Based Co-Design process.

**Topic guide radiographers**	**Topic guide patients**
**Background of role and experiences working in the service**, e.g.: can you describe a typical day's work in this service? What does a good day at work look like, and a frustrating one? What challenges do you encounter from a staff point of view?	**Treatment journey up to this point**, e.g., could you tell me a little about the first time you went in for radiotherapy treatment? What was your first impression? What stands out about meeting your radiographer for the first time?
**Perceptions of the patient experience**, e.g.: What do you think it is like to be a patient in this service? Which patient needs are met, which not? What are the things that really shape the patients' overall experience?	**Satisfaction with the radiotherapy service, including (1) the relationship with the radiotherapy staff and information provision**, e.g.: how satisfied have you been with the care you received during radiotherapy? How did you get on with the radiotherapy staff? Were there things that you were worried about but didn't know whether you could ask or not?
**Thoughts on improving communication in the service**, e.g.: how do you experience speaking with patients at the review meeting? Do you experience difficulties to talk at some length or in depth? What do you see as main priorities for improving communication with patients from the staff point of view? What do you think that patients would identify as priorities?	**… and (2) questions on the ‘best and worst bits’ of radiotherapy**, e.g.: what moments in the radiotherapy journey really shaped your overall experience? What were the crucial moments in talking with your radiographer?

Within the study department, the on treatment review clinic is undertaken by a team of three radiographers: a lead Radiographer who specializes in breast cancer and two radiographers who work clinically and undertake breast review as part of their role. It is a radiographer-led service where the role of the radiographer can include all aspects of the breast cancer patient journey from CT planning, review, consent, follow up as well as triaging problems post-radiotherapy. Female radiographers were involved in the co-design discussions, as male radiographers do not perform breast review in the study department.

#### Feedback Events

To prepare for the patient feedback event, topics were collated thematically and a short video was edited using fragments from the filmed interviews to reflect patient experience. In addition, a handout was designed that listed the identified “significant moments of interaction” with the service. To aid the discussion at the feedback event, this handout included questions from Synthesized Member Checking (Birt et al., 2016) to check whether the outputs matched the participants' experience and if they wanted to change anything. After discussion of the video and moments of interaction, an Emotional Mapping exercise^3^ was done at the feedback event, in which the moments of interactions were mapped out across the meeting room wall on a portable whiteboard. Participants were asked to map on a high-low scale how positive or negative that interaction with the service had been in their experience and to add words denoting their emotional experience. This map was discussed as a group with the researchers (MB, GH) and priorities were discussed for what to bring to the subsequent joint event with radiographers.

Prior to the radiographer feedback session, the researchers (MB and GH) framed core insights from the staff interviews as “Insight Statements”^4^ At the feedback session, these were presented on a handout with supporting quotes and discussed as a group to validate findings. Together, these “Insight Statements” were converted into “How Might We?” questions^5^, which were then mapped out on the wall display. The radiographers were invited to map their priorities as a group on a high-low scale, where higher on the wall indicated the topic was considered more important. A selection was made of topics to be addressed internally and topics to bring to the co-design work.

#### Staff-Patient Session

At the joint staff-patient session (3 h), participants first watched and discussed the patient experience video together. Card sorting was used to translate and narrow down topics identified in the feedback sessions to shared priorities to move forward. These priorities were then presented in the context of four significant interactions of patients with the radiotherapy service. In a brainstorm carousel, radiographer-patient pairs rotated through these settings to generate ideas to address the questions raised. To round up, the outcomes were discussed as a group.

#### Evaluation Co-design

The co-design process was evaluated through paper-based self-report surveys that are part of the online Experience-Based Co-Design toolkit^6^, distributed to the participants at the end of the session and collected ~1–2 weeks later via post, email or in person. The survey included seven questions about the patient experience video, discussing experiences with staff and patients, discussing priorities for the project, comfort with participation, any issues that were not discussed, and suggestions for improvements for future events. Participants could rate on a 5-point scale, from excellent to very poor, and space was provided for open-ended responses.

### Scaling Up: Iterative, Consultative Development

Following the co-design session, *N* = 24 national stakeholders were involved in the consultative, iterative development of a model for empathic communication and supporting training package. The aim was to “scale up” findings beyond the single center and include a national perspective in the development of the training, to ensure it reflects the reality of the wider service.

To build the model, co-design findings were mapped onto the KEPe Warm framework, which has been shown to reduce patient distress in primary care consultations (Little et al., 2015). Several resources were consulted for good practice on the format of the supporting training, including Draper and Silverman's framework for designing communication skills teaching sessions (Draper and Silverman, 1990) and reviews on communication skills training for healthcare professionals working with people who have cancer (Moore et al., 2018) and in the radiotherapy setting specifically (van Beusekom et al., 2019).

Starting with the communication model draft and storyboards with conversation scenarios, new training materials were developed and included throughout the iterative feedback process. Table 2 shows the number of participants providing feedback on each component, including representatives from patient support networks (*N* = 8), who all received treatment for breast cancer, but with varying treatment journeys, radiographers (*N* = 12) and other members of the National Society and College of Radiographers (SCoR) Research Advisory Group (*N* = 4), including education providers and service managers, experienced with a wide remit of cancers. Feedback was invited in the stakeholders' preferred format, using visual drafts and feedback grids (Interaction Design Foundation), including a group session and individual interviews (max 1 h) and via email. In addition, four SCoR members in managerial roles and two SCoR radiographers provided feedback on the overall training manual. When possible, feedback was incorporated into the materials directly. In case of practical barriers or conflicting recommendations, suggestions were discussed between authors MB and GH.

**Table 2:** Developed training materials and number of stakeholders who provided input.

**Description**	Radiographer feedback^*^	Patient feedback^*^
**KEW model** Model for empathic communication: Know, Encourage, Warmth	*n* =12 (focus group; individual feedback–survey)	*n* = 6 (individual feedback–email/in person)
**Storyboards/trigger videos** Scenarios for difficult conversations between radiographers and breast cancer patients. Videos for use in training to trigger discussion on personalized strategies	*n* = 12 (focus group; individual feedback–survey)	Storyboards: *n* = 5 Trigger videos: *n* = 1 (individual feedback–email/in person)
**Fear of Cancer Recurrence handout** Strategies for (1) gauging concerns, (2) encouraging conversation, (3) addressing fears of recurrence. Prompt to reflect on who patients can talk with within their service	*n* = 1 (individual feedback–email)	*n* =3 (individual feedback–email/in person)
**Simulated Patient Scenario** Detailed background for a fictional patient receiving radiotherapy for breast cancer, to use for role-play with simulated patient	*n* = 1 (individual feedback–email)	*n* =1 (individual feedback–email)
**Informed Consent form** Sheet and instructions for radiographers to use with patients for training audio-recording	*n* = 2 (individual feedback–survey)	*n* =2 (individual feedback–email)
**Patient Experience Exercise** Sheet detailing patient interactions with the radiotherapy service and common emotions. Prompts to reflect on how to apply the KEW principles at each stage and service-specific considerations, e.g., the waiting area	*n* = 1 (individual feedback–email)	*n* =1 (individual feedback–email)

* *Some stakeholders provided feedback on more than one component. For anonymity purposes, numbers have been clustered. Radiographers include: n = 10 Radiographers from SCoR and n = 2 from ECC. Patient Representatives include members from Independent Cancer Patients Voices, Yorkshire Cancer Patients Forum, and Maggie's Center*.

## Results

### Patient Experiences

**Figure 2:**
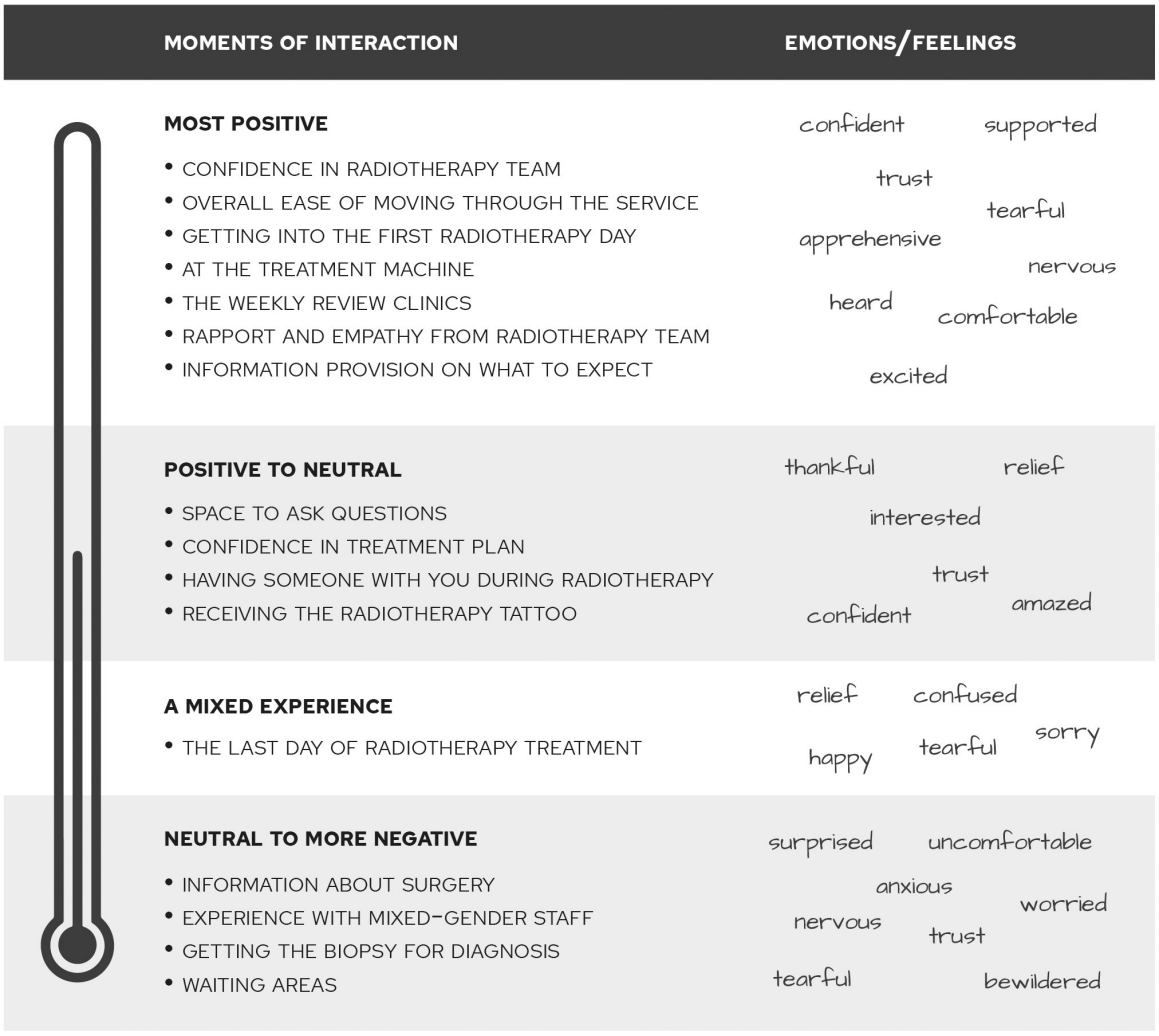
Moments of interaction with emotion words as ranked in the patient feedback session.

The patient interviews and feedback session resulted in an overview of interactions with the radiotherapy service that participants felt shaped their experience in a significant way. Figure 2 gives an overview of these interactions and a summary of what emotional states participants associated with them. Overall, participants felt mostly positive to start radiotherapy, especially after consideration of some more negative experiences leading up to this stage, such as the anxiety of waiting for the biopsy results and feeling scared about surgery. The overall ease of moving through the service and from appointment to appointment was rated highly.

However, participants also expressed to have felt some apprehension at several moments. One of those was being under the treatment machine. As a participant commented on her experience: ***“In the end, it's you and the machine, and you're never going to get to the point where you feel friendly toward this machine, although you know that it's helping you. It's a sort of bizarre experience.”** (Patient (Pat) 4, female, age 70)*.

Another moment where patients felt apprehensive was going in for the first day of radiotherapy treatment. A participant described: ***“I felt quite lost actually first. And then it was fine, I met the girls who were taking me through and I just got on with it.”** (Pat 3, female)*. The waiting areas, which were rated as a relatively negative moment of interaction, also played a role in this context: ***“I think the first time coming in, it would be nice to have someone come meet you at the door. You do get kind of confused and lost if you've never dealt with this before.”** (Pat 3, female)*. The waiting areas were also described as ***“a little bit bleak. I think it could be a little more uplifting”** (Pat 2, female, age 70)*.

Participants described some unexpected moments that left a significant impression, such as receiving the radiotherapy tattoo: ***“I'm needle-phobic and that was quite a shock. I knew I was having them, I don't know how I thought they were actually going to do it.”** (Pat 1, female, age 51)*. Also, encountering a male member of staff at the treatment machine was mentioned as a bit of a surprise: ***“There was a male, and I wasn't uncomfortable that's the wrong word, but I do prefer to have two female radiographers. You are feeling very stressed and anxious and it's a very intimate thing to do.”** (Pat 2, female, age 70)*

The support, trust and confidence from the radiotherapy team that patients felt was rated as the most positive interaction. As a participant describes: ***“You get the feeling while they are doing that that they are very much in control of what they are doing. They know exactly what's going on. And that's very reassuring. That's what you want. What you don't need when you're going through this is anyone who feels the slightest bit nervous or uncertain about what they are doing, because that immediately transfers onto you.”** (Pat 2, female, age 70)*. In the context of the rapport and empathy they experienced from the radiotherapy team, a participant emphasized the importance of ***“just being treated like an individual, rather than another patient number.”** (Pat 4, female, age 70)*

A cluster of significant interactions focused on this type of support, including the importance feeling that there was enough space to ask questions despite the full waiting room, good information provision to establish a sense of what is going on and having the support of a partner or family member. The weekly review clinics in particular were mentioned as an opportunity for more in-depth conversations. As a participant describes: ***“[The radiographer] used the expression of radiotherapy as a sort of insurance policy, which I found very reassuring. (…) Toward the end of the second [review] I was able to ask her the key question “what would you do if this did do the worst and spread to say my bones?” (…) She talked a little bit about that with me and said that people who have cancer of the bones can still live a pretty good life. It probably would be good if there were built in more opportunities like the review where it was just you and one other person being able to talk about you as an individual.”** (Pat 4, female, age 70)*. As a result of the value of this support, participants indicated to have experienced mixed emotions around leaving the service: while it was a relief to be finished, it was also a confusing time to have to leave this support system.

The radiotherapy review session and the role of the person leading it as “trouble shooter” was considered essential and the topic of “space to ask questions” was identified by the patient participants as a priority for co-design. As a participant described: ***“It felt possible to ask questions. It was more my awareness that these were people who were quite pressed for time and there were a lot of other people in the waiting room.”** (Pat 4, female, age 70)*.

### Radiographer Experiences

**Figure 3:**
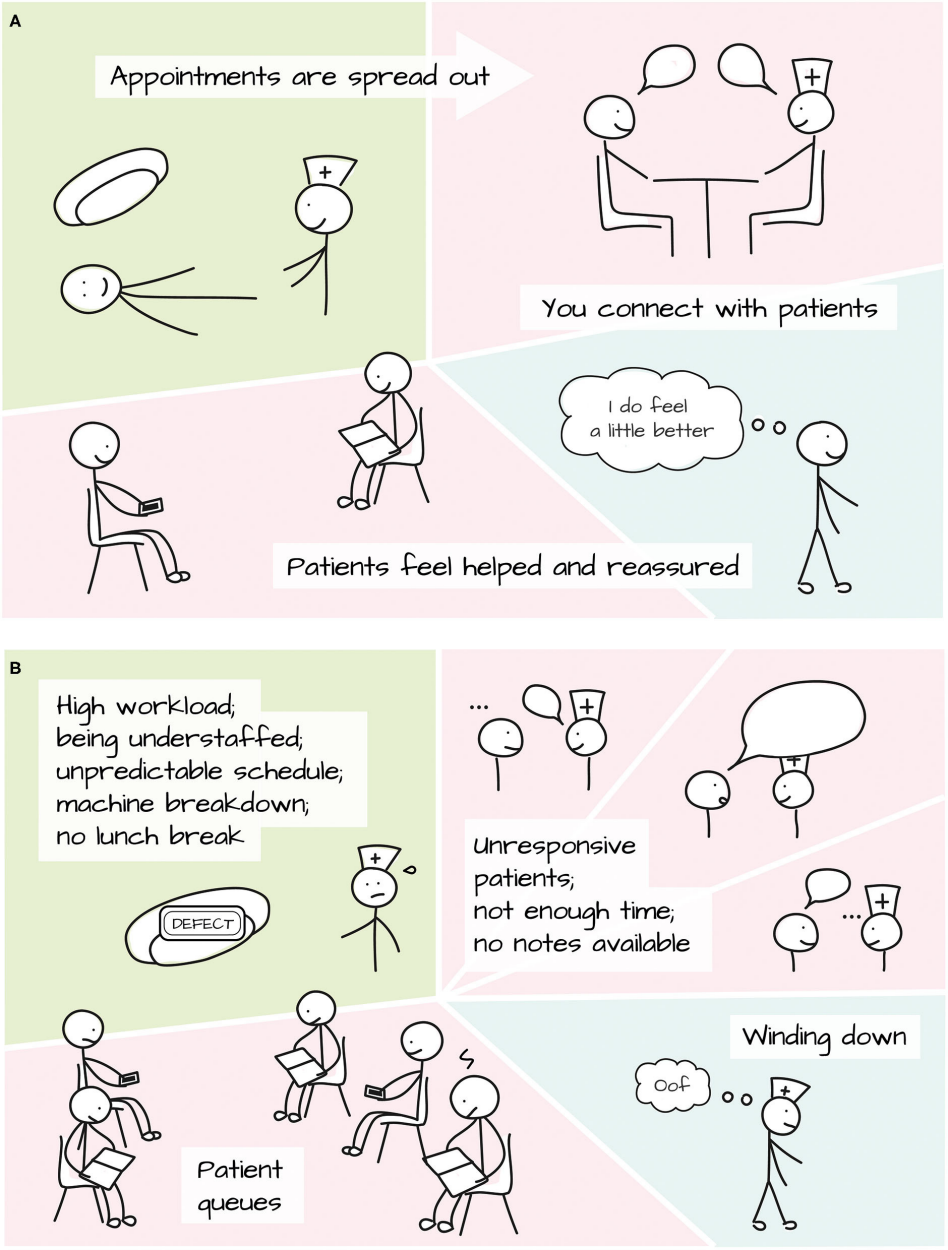
A **(A)** good and **(B)** difficult working day for the radiographers.

The following Insight Statements relating to the radiographers' experience resulted from the radiographer interviews and feedback session, captured by six themes: (I) A good day at work, (II) Logistical barriers, (III) Supporting patients, (IV) Review Clinics, (V) Support with communication, and (VI) Team support. Figure 3 illustrates a good and difficult working day for the radiographers.

Theme I: A good day at work

1) The RT team particularly enjoy their workday when they feel that patients leave the service feeling helped and reassured: ***“When you feel like you've helped somebody. And (…) that they leave the appointment and they're like “Oh, I feel so much better, you really reassured me about that.””** [Radiographer (Rad) 1]*
2) On a good day in the clinic, patient appointments are nicely spread out throughout the day: ***“… that we have enough spaces in between and a lunch break, really.”** (Rad 2)*

Theme II: Logistical barriers

3) The heavy workload and understaffing can be tiring and feel unmanageable for the radiotherapy team: ***“Sometimes we're down to one person covering the service (…). Doing all these patients on your own for a long period isn't sustainable.”** (Rad 1)*
4) The unpredictable patient schedule can lead to queues and consequently to frustrations for patients and radiographers feeling “on edge” because of time constraints: ***“We're at the liberty of the treatment machine. So if they are running an hour late, we are then an hour late.”** (Rad 3)*
5) It can be particularly difficult to lead a review session when patient notes are not available before going into the clinic: ***“Quite often, the notes won't be there (…). I feel like you're at a massive disadvantage because you're going blind into a review.”** (Rad 2)*

Theme III: Supporting patients

6) Overall, the RT team think patients are pleased with the “caring and careful” staff. Most practical/information needs are met, as well as many of the emotional needs: ***“I had about three or four different patients who all said to me (…) the staff were so caring and interested in them as a person.”** (Rad 1)*
7) The wide range of patient needs and communication styles can be challenging to deal with, such as patients who had a complex treatment journey and who may feel angry, embarrassed or scared and challenge offers for help. Some patients may not be very talkative, while others would like to talk more than there is time available.
  - ***“She said she wasn't coping, but was unwilling to accept any help”*** *(Rad 1)*
  - **“For some people, their fear presents as anger”** (Rad 4)
  - **“Often you are kind of having to end the conversation”** (Rad 3)
8) The RT team see that on the first day and last day of treatment patients may need extra support: ***“I still think it must be very daunting and scary for them (…) not fully understanding exactly what's going to go on.”** (Rad 3)*

Theme IV: Review clinics

9) Review clinics are essential to offer psycho-social support as the time with patients at the machine is not sufficient to cover such topics: ***“You do get more time to actually sit down (…), you can start to explore different things that are going on.”** (Rad 2)*
10) The repeated review sessions are perceived as helpful to **build rapport** with patients: ***“You do build up a rapport with patients (…). They've maybe thought from one week to the next about what they want to ask about whatever it is.”** (Rad 1)*

Theme V: Support with communication

11) The RT team is keen to receive communication training to boost confidence in offering (mainly psycho-social) support, in particular with questions such as: how to make a patient feel valued as a person, deal with challenging behavior, determine needs, address them concisely, prompt non-talkative patients, round up the conversation in a respectful manner, refer patients with emotional concerns, address topics such as worries about treatment or fear of recurrence. ***“The thing we're not trained for though is the emotional side. That's certainly an area that (…) I would like to (…) be able to deal with.”** (Rad 3)*
12) The RT team support also each other with communication strategies for more challenging patient cases: ***“Say that there is a particularly difficult patient or a difficult situation with a patient, we always sit and chat about it.”** (Rad 3)*

Theme VI: Team support

13) It is important to have the opportunity for support and decompression after difficult cases on a day-to-day basis: ***“If you can relate to that–it's been a horrible situation–then that is actually quite hard, and you can then take that home.”** (Rad 4)*

The radiographers identified the issue of offering psycho-social support as the priority to bring to the co-design session. Items relating the logistics and workload of the service were taken forward by the radiographers to discuss and address internally.

### Shared Priorities and Solutions

At the joint patient-staff event, after watching the patient experience video, radiographers and patients collaboratively narrowed down their shared priorities to: (I) making patients feel valued as a person, (II) addressing topics such as worries about treatment or fear of recurrence, and (III) supporting patients with space to ask questions. Additionally, it was agreed to include the (IV) less positively rated experience encountering male staff at the treatment machine and the waiting area in the brainstorm session. These themes were presented in the context of significant settings as identified: the first and last day of treatment, at the treatment machine, the review clinic, and the overall journey through the service, including the waiting area.

**Figure 4:**
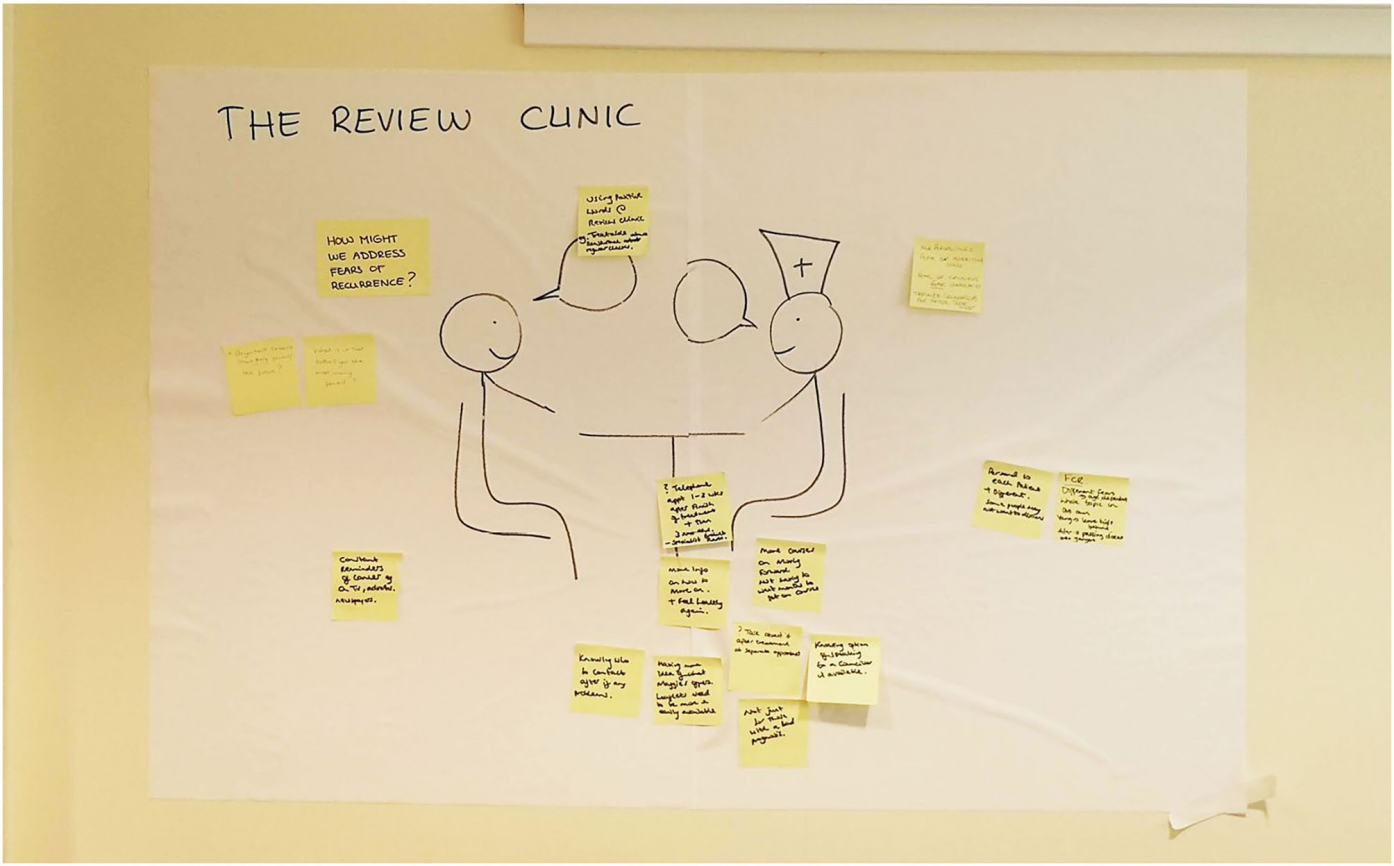
One of the idea generation roulette “stations” at the joint staff-patient session.

A recurring theme in the idea generation process (Figure 4) was supporting patients with knowing what to expect. For example, to reduce apprehension at the start of treatment, the group suggested to revisit the day-to-day logistics and reiterate information, including reassurances, over the first couple of visits. This was considered a helpful addition to written information and to support understanding from family members as well. Personal contact such as “***being greeted and checked you're okay***” in this context was identified as essential and could be complemented by having someone meet the patient at the reception.

Checking the patient's understanding of what is going to happen was advised to avoid catching them off-guard in a situation where they already feel vulnerable. For example, regarding the issue of being helped by a male member of staff, the group suggested it would already make a difference to simply know this in advance, before they undress for the treatment machine. Knowing what to expect was also a key theme in addressing the topic of space to ask questions. In addition to the identification of settings that provide a good opportunity for questions, such as at the treatment machine with the radiographer and at the review clinic, the group recommended indicating when time would be available if a longer conversation was not possible, for example by offering an additional appointment to respond to the patient's questions.

Next to this “deferring” strategy, “referring” to other staff was recommended as a strategy to help manage patient questions. In an ideal situation, there would be a dedicated person in the service to deal with appointment questions and provide emotional support also outside of the review clinics. A smooth “handover” was also recommended to support patients with apprehension on the last day of treatment. The group agreed that the focus should be on creating awareness of existing support services, such as the breast care nurse, Maggie's Centre programmes and counseling services. It was also suggested to have a telephone follow-up with the radiotherapy team 1 or 2 weeks after treatment completion and again after 3 months, to be able to talk with a “familiar face.”

To support patients with feelings of fear or cancer recurrence during their time in the radiotherapy service, it was recommended to use positive words throughout conversations, such as “treatable” in relation to the cancer and to give reassurance about regular checks. The group also agreed on being direct in addressing this topic, for example by asking “Do you have concerns about going forward/the future?” or “What is it that bothers you most moving forward?” It was acknowledged that some patients may not want to discuss these feelings; however, staff and patients agreed that this questioning should not be limited to people with poorer prognoses.

Suggestions for the waiting area included to make it more uplifting by removing medical pictures and designing what patients would consider, in consultation, a positive and bright space. A layout was recommended that encourages conversations between patients. In addition, some distractions could be added such as perhaps a TV.

### Co-design Evaluation

Evaluation of the co-design process showed that talking about and sharing the different experiences of staff and patients was rated by radiographers as “excellent” (no free responses) and by patients as “good” to “excellent.” This exchange was appreciated by a patient who commented that ***“this felt worthwhile, as the needs of patients met within the realities of the radiographers' jobs. As always, time and resources are crucial”** (Pat 4). Two* patients noted that the patients' shared mostly positive experiences, e.g.: ***“I found this very useful, although I did feel the patients had all had similar, very positive experiences”** (Pat 1)*.

Seeing the patient film was rated consistently by both parties as “good” to “excellent.” Patient participants reported that the video represented their experiences well and enjoyed watching it: ***“The film was carefully edited, so that there was a coherent thread running through it. People clearly felt sufficiently at ease to give their ideas and experiences at each point”** (Pat 4)* and ***“I enjoyed hearing the other women's experiences–I thought the film was fair, well-constructed and balanced, with everyone having a say”** (Pat 1)*. A radiographer commented that ***“what was said was what I hoped the patients thought, but it was good to hear”** (Rad 3)*.

Discussing and deciding the priorities that would be worked on and improved was rated as “excellent” by the radiographers (no free responses) and “good” to “excellent” by patients. A patient mentioned that despite the positive tone of the experiences ***“we were still able to find areas to improve and work on”** (Pat 1)*. A patient said that they ***“felt privileged to be part of the changes that could be made, big or small”** (Pat 42)*. Again, the exchange between staff and patients was valued: ***“this section was focussed and useful. It was good to have a member of staff in the groups, so it was not dominated by patients' experiences”** (Pat 4)*. With regards to the outcomes of the priorities, a patient expressed her hope that ***“perhaps this will help to create a follow-up service, required for future patients' mental health issues linked to breast cancer”** (Pat 2)*.

Radiographers rated how comfortable they felt participating in the event and their ability to contribute their own thoughts and experiences as excellent (no free responses) and patients as “good” to “excellent.” A patient participant described the atmosphere as ***“welcoming (…) we all got to have our say”** (Pat 1)* and others described their participation as ***“very positive”** (Pat 2)*, as well as ***“comfortable”*** and feeling ***“valued as a contributor”** (Pat 4)*.

Radiographers did not feel that there was anything that they did not get a chance to say that they had wanted to contribute and most patients agreed. One patient added that she would have liked to learn more about how the radiographers envisage their ideas being put into practice and whether the organization will be flexible enough to allow this: ***“communication training will take time, and will involve commitment from staff, meaning time has to be made available”** (Pat 4)*.

Points for improvement of the co-design process were ***“more time***” *(Rad 2)* from the radiographers' point of view. With respect to the positive experiences in the room, a patient suggested that ***“it might be useful to hear from patients whose experience was less positive–it might broaden the discussion a little”** (Pat 1)*. Another patient mentioned that sharing experiences can sometimes ***“result in a loss of focus at points***” *(Pat 4)*. Overall, both radiographers and patients rated the organization of the co-design process as good to excellent.

### KEW: Know, Encourage, Warmth

**Figure 5:**
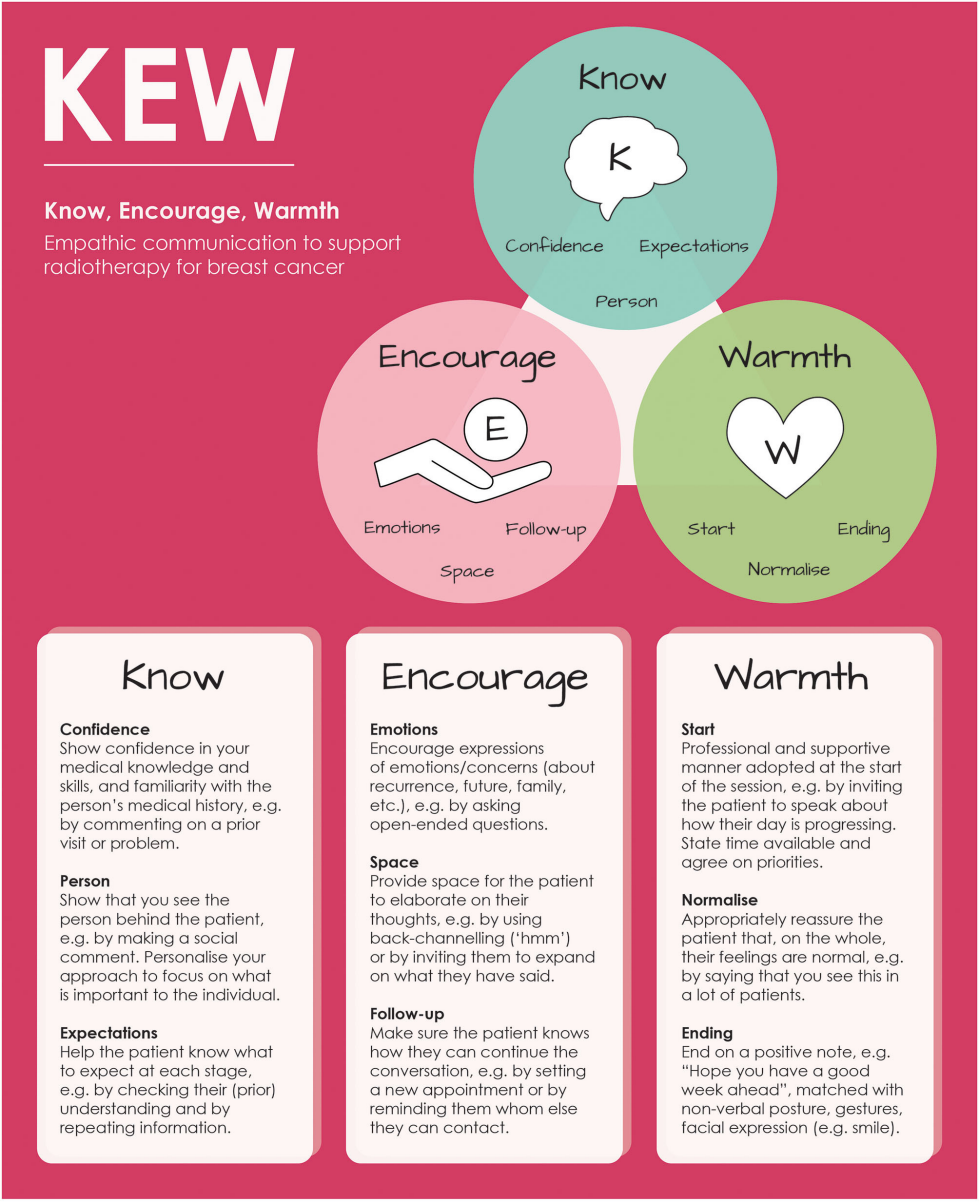
The Know, Encourage, Warmth (KEW) model for empathic communication.

Most priorities identified in the co-design session related directly to communicative behaviours, i.e., making patients feel valued, addressing difficult topics, and providing space. Mapping these priorities and themes from the Experience-Based Co-Design process onto the KEPe Warm model (Little et al., 2015) resulted in a model for empathic communication, KEW, for Know (Confidence, Person, Expectations), Encourage (Emotions, Space, Follow-up), and Warmth (Start, Normalize, Ending), as clarified in Figure 5.

Feedback from national radiographer and patient representatives indicated that overall, the model was considered to be clear and concise and its components important and relevant. A radiographer commented: ***“I especially like warmth. It's a caring profession and an aspect that's important to me. Knowledge is also essential and having confidence when discussing information with patients***.” Also patients' feedback on the model was positive: ***“The KEW model accurately represents the good parts of the patient experience: the empathy, showing the person behind the patient by making social comments, giving realistic expectations and realistic timeframes***.**”**

Some suggestions for changes could be directly included in the model, such as a radiographer's comment on the importance of a personalized approach for patients (***“No mention of individualizing/personalize as well as normalizing”***) that led to the inclusion of this topic under the subcategory “Person” within the theme “Know.” A patient's remark about the risk of normalizing feelings that were *not* the usual reaction ***(“Under normalize important to also insert wording to cover situations that are not the usual reaction”***), led to rephrasing of the relevant sentence under the subheading “Normalize” within the theme “Warmth.”

Other feedback could be incorporated in the training manual that was developed alongside the KEW model, such as the advice to broaden the use of the model to be implemented throughout the radiotherapy setting and the suggestion to address barriers radiographers may experience to start potentially difficult conversations with patients. Following repeated suggestions, pragmatic advice with specific examples on how to achieve KEW strategies were included, and practice-based examples were generated in feedback rounds by asking participants to supply responses to conversation scenarios described in the storyboards. This led to a database of examples that can be used in the training by the facilitators. Another category of feedback recommended to encourage management support, which was addressed by including managerial staff in the feedback rounds and including links to national strategic cancer frameworks in the training package.

Feedback on the other training components as described in Table 2 consisted of acknowledgment of their relevance for clinical practice and relatability to the diversity of patient experiences, with detailed suggestions on how to match the flow of medical procedures, use of language, and patient experience even more accurately. To illustrate, for the simulated patient scenario, a patient representative commented: ***“…“surgery “smooth” except for infection and redressing”–most of us as patients would think this as major not just a hiccup***,**”** while a radiographer commented that ***“the time delay between biopsy and seeing the surgeon is more realistically 2 weeks***.**”** These changes were then made to the materials accordingly.

In consultation with radiographers, it was agreed that two half day sessions would be a suitable format for the resulting training workshops, as described in Table 3.

**Table 3:** Outline of KEW training workshops.

**Session 1**	**Between sessions**	**Session 2**
Activation of prior knowledge *Learner- and patient centered exercise; reflection on prior knowledge* Trigger videos, handout	Audio-record two review sessions with patients	Warm-up exercise *Activation of prior knowledge* (Trigger video, handout)
Introduction of KEW model *Definition of skills* (Presentation with slides, KEW handout)		Reflection on audio-recordings *Experiential Learning, ALOBA*
Fears of Cancer Recurrence *Reflection on prior knowledge, definition of skills* (Trigger video, FCR handout)		KEW in the wider service Patient experience exercise handout)
Role play with Simulated Patient *Experiential learning, Agenda-Led Outcome Based Analysis (ALOBA)* (Scenario, handout)		
Setting personal goals		

## Discussion

This study describes the development of a concise model for empathic communication in radiotherapy for breast cancer: KEW, for Know, Encourage, and Warmth, building on service user-experiences of radiographers and breast cancer patients. The linked communication skills training provides radiographers with theory and tools on how to provide space and foster empathic communication with patients throughout their treatment pathway, with a focus on key interactions such as the first and last day of treatment and the review clinic. The training also enables radiographers to develop personalized strategies to discuss difficult topics with patients, such as fear of cancer recurrence.

Emotionally, radiotherapy is known to consist of a series of ups and downs for patients (Humphris et al., 2019). The Experience-Based Co-Design process helped capture what factors contribute to a positive patient journey, including the support, confidence, and personable approach from the radiotherapy team to help reduce apprehension at key moments of interaction with the service. From this study, it is apparent that radiographers are keen to receive training to help increase their own confidence in how to best offer emotional support while being realistic about time constraints, to move beyond the feeling of potentially “opening a can of worms” when starting a difficult conversation.

It is already known that encouraging emotional cues and responding to these appropriately can improve quality of medical consultations (Little et al., 2015), which is reflected in existing communication training for the radiotherapy setting (Timmermans et al., 2006; Halkett et al., 2012, 2013, 2018; Hollingworth et al., 2013). The outcomes of the co-design approach suggest the need for a more encompassing approach to improving the patient experience: i.e., not only focussing on the quality of communication and information provision during a single conversation, but ensuring that patients are not “left hanging” between these formally organized interactions and are supported throughout their journey within the service by “referring” or “deferring” conversations. This integrated approach is reflected in the training programme, along with a focus on realistic and timely expectation management in patient communication, described by some as being part of the empathic process (Underhill et al., 2014). The review clinic, which in the UK patients have the opportunity to attend to discuss their treatment and general reactions with their radiographer (Cameron et al., 2008), was identified as a key opportunity for more in-depth conversations and to help the build rapport to foster the warm and personal approach to communication that helps to reassure patients.

The Experience-Based Co-Design approach was valued by patient and staff participants. The visual tools, in particular the recorded patient stories, and interactive exercises effectively triggered the partnership synergy required for successful collaboration (Lasker et al., 2001; Bate and Robert, 2007). However, this rigorous process included only a small number of, all female, patient participants who were relatively positive about their experience. As a result, the initial co-design process allowed the research team to capture good practice. To improve generalisability, the findings were scaled up using consultative input from national radiotherapy and patient representatives. The number of participants for each step were kept small for several reasons: (1) the co-design process in particular required intensive facilitation and guidance to ensure that all involved felt comfortable with their participation (2) the practical aspect of the timeline of the development process, and (3) reducing the burden for research participants–the research team aimed to encourage active participation for in-depth contributions, which is not a small ask both in terms of offering sufficient guidance and from the participant perspective. Previous experience with stakeholder-involvement showed that the iterative aspect to the development process is a key factor to target the outcome to the needs of the end-users (van Beusekom M. et al., 2018). The described process led to invaluable insights on how to take a tailored approach both with respect to patient communication and to the communication skills workshops for radiographers, to be able to reflect the diverse range of experiences and services.

The main aim of the collaborative process was not to improve a single service, but to develop a wider applicable intervention. Participants were aware of this, which may have influenced their decision-making process regarding prioritization of topics. However, the participant-led discussions included a wide range of topics, including site-specific concerns around the waiting area. This topic, along with other site-specific suggestions have been included in the training package as points of reflection for the overall patient experience. In addition, specific suggestions for improvement were also fed back to managerial staff of the service where the co-design was conducted.

### Conclusions

In conclusion, the support from the radiotherapy team is a significant contributor to a positive patient experience. The Know, Encourage, Warmth (KEW) model and training offer a concise framework and tailored approach to help radiographers support breast cancer patients with emotional concerns throughout their radiotherapy journey. The use of single-site Experience-Based Co-Design in combination with consultative input from national stakeholders proved useful to capture good practice and optimize wider generalisability to help standardize quality in communication across radiotherapy services. Delivery and evaluation of the communication skills training is warranted to determine effectiveness to prepare for roll-out on a wider scale and to examine the potential for use of the framework across a range of cancer sites as well as other staff working with cancer patients.

## Data Availability Statement

The original contributions presented in the study are included in the article/supplementary material, further inquiries can be directed to the corresponding author.

## Ethics Statement

The studies involving human participants were reviewed and approved by London–Surrey Research Ethics Committee. The patients/participants provided their written informed consent to participate in this study.

## Author Contributions

GH designed the overall study. MB developed the co-design methodology and led the data collection. JC and CB were essential in the implementation. EB and RH in the involvement of national patient and radiographer representatives. MB and GH undertook the analysis and wrote the first draft. All authors contributed to the design and assisted with the interpretation of the findings and drafts and read and approved the final manuscript.

## Conflict of Interest

The authors declare that the research was conducted in the absence of any commercial or financial relationships that could be construed as a potential conflict of interest.

## Footnotes

^1^The Point of Care Foundation. EBCD: Experience-based co-design toolkit. Available from: https://www.pointofcarefoundation.org.uk/resource/experience-based-co-design-ebcd-toolkit/

^2^The Point of Care Foundation. EBCD: Experience-based co-design toolkit. Available from: https://www.pointofcarefoundation.org.uk/resource/experience-based-co-design-ebcd-toolkit/‘

^3^The Point of Care Foundation. Running the Patient Feedback Event [Available from: https://www.pointofcarefoundation.org.uk/resource/experience-based-co-design-ebcd-toolkit/step-by-step-guide/11-running-patient-feedback-event/

^4^IDEO.org. Create Insight Statements [cited 2019 09]. Available from: http://www.designkit.org/methods/62

^5^IDEO.org. How Might We [cited 2019 09]. Available from: http://www.designkit.org/methods/3

^6^The Point of Care Foundation. EBCD: Experience-based co-design toolkit. Available from: https://www.pointofcarefoundation.org.uk/resource/experience-based-co-design-ebcd-toolkit/

## Abbreviations

FCR: Fear of Cancer Recurrence
EBCD: Experienced-Based Co-Design
RT: radiotherapy
Pat: patient
Rad: radiographer.
